# In-vitro Assessment of the Proliferation and Apoptosis of Thyroid Cancer Cells Using Valproic Acid and Retinoic Acid Alone and in Combination with Etoposide and Epirubicin

**DOI:** 10.5812/ijpr-163580

**Published:** 2025-08-16

**Authors:** Ramin Ebrahimi Kiasari, Ghazaleh Ghavami, Soroush Sardari

**Affiliations:** 1 ِDrug Design and Bioinformatics Unit, Department of Medical Biotechnology, Biotechnology Research Center, Pasteur Institute of Iran, Tehran, Iran

**Keywords:** Papillary Thyroid Cancer, Valproic Acid, Retinoic Acid, Etoposide, Epirubicin

## Abstract

**Background:**

Thyroid cancer remains a significant global health concern, necessitating the development of more effective treatment strategies.

**Methods:**

This study investigated the therapeutic potential of valproic acid (VA) and retinoic acid (RA), both as single agents and in combination with conventional chemotherapeutics etoposide (Et) and epirubicin (Ep), using in vitro models of thyroid cancer. The research employed two representative cell lines: B-CPAP (poorly differentiated thyroid carcinoma) and SW-1736 (anaplastic thyroid carcinoma). Through comprehensive experimental approaches, including MTT viability assays, flow cytometry-based apoptosis and cell cycle analysis, and scratch wound migration assays, we systematically evaluated the compounds' effects.

**Results:**

The results revealed distinct pharmacological profiles: The RA demonstrated superior cytotoxicity with significantly lower IC_50_ values (3.01 µg/mL for B-CPAP and 1.83 µg/mL for SW) compared to VA (407.29 µg/mL and 584.32 µg/mL, respectively). Importantly, RA exhibited strong synergistic effects when combined with Et/Ep [Combination Index (CI) < 1], while VA showed primarily additive or antagonistic interactions (CI ≥ 1). Mechanistically, RA combined with low-dose Et/Ep (1/5 IC_50_) significantly enhanced early apoptosis rates (P < 0.05) and induced S-phase cell cycle arrest, effects not observed with VA combinations. In migration assays, RA completely inhibited cancer cell movement (100% inhibition), outperforming VA's partial inhibition (40 - 70%).

**Conclusions:**

These findings collectively demonstrate RA's potent anticancer activity and its ability to synergize with conventional chemotherapeutics, highlighting its potential as a promising candidate for combination therapy in thyroid cancer treatment. The study provides compelling preclinical evidence supporting further investigation of RA-based therapeutic strategies through advanced preclinical studies and clinical trials.

## 1. Background

According to the WHO GLOBOCAN 2020 database, thyroid cancer ranks as the ninth most common cancer globally. While it can occur at any age, it is most frequently diagnosed in individuals in their early 50s and is the most prevalent malignancy among individuals aged 16 - 33 years (1). Thyroid cancers are classified into three major types: Differentiated thyroid cancer (including papillary, follicular, and oncocytic subtypes), medullary thyroid carcinoma (often associated with MEN2 syndrome), and anaplastic thyroid cancer, which has a particularly poor prognosis and often evolves from differentiated forms (2).

A critical barrier to successful treatment is tumor heterogeneity and drug resistance, both intrinsic and acquired. These factors reduce the efficacy of monotherapy approaches and highlight the necessity for combination therapies that may overcome these limitations (2-4). Combination therapy, by employing agents with different mechanisms of action, is less likely to encounter cross-resistance and has the potential to induce deeper and more sustained remissions (5, 6). Despite promising preclinical data, translating combination therapy into clinical success remains challenging due to trial design limitations and patient recruitment barriers (5-7).

Common chemotherapeutics for thyroid cancer include doxorubicin, paclitaxel, docetaxel, cisplatin, etoposide (Et), and epirubicin (Ep). However, their use is limited by off-target toxicities, such as cardiotoxicity, nephrotoxicity, neurotoxicity, and gastrointestinal effects, particularly in elderly patients (3-7). Thus, minimizing toxicity while maximizing therapeutic outcomes through rational drug combinations remains a key research focus.

Retinoic acids (RA), active derivatives of vitamin A, modulate cellular growth and differentiation via nuclear receptors RAR and RXR. The RA has been shown to induce redifferentiation in thyroid carcinoma cell lines, increasing the expression of thyroid-specific genes like the sodium-iodide symporter (NIS) and enhancing iodide uptake (8-10). Clinically, RA improves iodine uptake in previously refractory patients and demonstrates antiproliferative and pro-apoptotic effects, while generally being well-tolerated (8-13).

Valproic acid (VA), traditionally used to treat epilepsy, has gained attention for its anticancer properties. It acts as a histone deacetylase (HDAC) inhibitor, affecting gene expression, cell cycle arrest, and apoptosis. The VA has shown efficacy in thyroid cancer models and possesses anti-angiogenic effects (14, 15). It has also been linked to the modulation of c-Met and hepatocyte growth factor (HGF) signaling pathways, both of which are implicated in cancer proliferation and metastasis, although their interaction with VA remains understudied (16). Despite the independent anticancer activities of RA and VA, few studies have assessed their combined effects with standard chemotherapeutic agents in thyroid cancer.

## 2. Objectives

This study aims to fill that gap by investigating the in vitro effects of RA and VA — alone and in combination with Et and Ep — on thyroid cancer cell proliferation, apoptosis, necrosis, and migration. The results are expected to contribute valuable insights into the development of more effective, less toxic combination therapies for thyroid cancer.

## 3. Methods

### 3.1. Cell Culture

Three cell lines were utilized: B-CPAP (poorly differentiated thyroid carcinoma, ATCC CRL-1803), SW-1736 (undifferentiated anaplastic thyroid carcinoma, IBRC C10311), and Hu02 (normal human foreskin fibroblast, IBRC C10309), all sourced from the Iranian Biological Resource Center (Tehran, Iran). These were cultured in RPMI 1640 medium supplemented with 10% fetal bovine serum (FBS, Biosera, France) and 2 g/L HEPES buffer at pH 7.4, maintained in a humidified incubator with 5% CO_2_. For assays, cells were seeded at 1×10^4^ cells/cm^2^ in 96-well plates for MTT and at 1×10^5^ cells/cm^2^ in 24-well plates for apoptosis, cell cycle, and migration studies. Each experiment was performed in triplicate for statistical accuracy.

### 3.2. MTT Cell Viability Assay

The cytotoxicity of VA and RA, alone and in combination with Ep and Et, was evaluated using the MTT colorimetric assay (Sigma-Aldrich, Germany) (17). After 24-hour drug exposure, MTT reagent (20 μL, 5 g/L) was added and incubated for 4 hours. The formazan crystals were solubilized with 100 μL DMSO, and absorbance was measured at 545 nm using a multiwell ELISA reader (Organon Teknika, Netherlands).

### 3.3. Apoptosis and Necrosis Detection (Flow Cytometry)

For apoptosis analysis, B-CPAP and SW-1736 cells were incubated with treatments for 48 hours. Non-adherent cells were collected, and adherent ones were detached with trypsin-EDTA. Annexin V-FITC and propidium iodide (PI) staining was performed using IQ Products (Netherlands) according to the manufacturer’s protocol (18). Flow cytometric analysis was conducted with the CyFlow system (Partec, Germany).

### 3.4. Cell Cycle Analysis

To analyze the effects of drugs on cell cycle distribution, treated cells were fixed with 70% ethanol at -20°C for 6 hours. Following PBS washes, cells were stained with a solution containing 0.1% Triton X-100, 0.5 mg/mL RNase A (Sinaclon, Iran), and 0.025 mg/mL PI (Sigma-Aldrich, Germany). Samples were analyzed via flow cytometry (CyFlow) (19).

### 3.5. Wound Healing (Scratch) Assay

Cell migration was evaluated by performing a scratch assay on confluent B-CPAP and SW cells grown in 24-well plates. Scratches were created using pipette tips, and cells were treated with compounds. After 48 hours, migration was assessed via microscopy and quantified using the formula (20): Migration % = [Scratch distance (initial time) - Scratch distance (after 48 hours)]/Scratch distance (initial time)] × 100.

### 3.6. Statistical Analysis

All experiments were conducted in triplicate (n = 3), and the obtained data were analyzed using GraphPad Prism 5.0 (GraphPad, La Jolla, CA, USA) to determine IC_50_ values, including nonlinear regression equations with 95% confidence intervals, as well as mean ± standard error of the mean (SEM). Statistical analysis was performed using one-way ANOVA followed by Tukey's post hoc test in GraphPad Prism 5.0. A P-value of less than 0.05 was considered statistically significant. The Combination Index (CI) method was applied. The CI values were computed using ComboSyn software (ComboSyn Inc., NY, USA), based on the Chou-Talalay CI theorem. This approach provides a quantitative measure of drug interactions, where CI < 1 indicates synergism, CI = 1 denotes an additive effect, and CI > 1 reflects antagonism.

## 4. Results

### 4.1. The Impact of Single and Combined Drug Concentrations on Cell Proliferation

The study investigated the effects of single and combined drug concentrations (VA, RA, Et, and Ep) on thyroid cancer cell lines (B-CPAP and SW) through multiple experimental approaches. Initial assessment using the MTT assay, as presented in Table 1, demonstrated that VA and RA exhibited toxicity against B-CPAP and SW cells when administered individually. When these compounds were combined with Et and Ep, synergistic effects were observed, as evidenced by a CI < 1. The combination of RA with Ep and Et demonstrated strong synergistic effects (CI < 1) in B-CPAP and SW cancer cell lines, with the lowest CI values observed at 0.02237 and 0.02476, respectively. In contrast, VA primarily showed additive or antagonistic effects (CI ≥ 1). Notably, in normal Hu02 cells, all combinations containing RA and VA exhibited significant antagonistic effects (CI > 1), indicating these compounds' selective toxicity against cancer cells. These findings confirm RA's superiority as an adjunctive agent in thyroid cancer chemotherapy.

**Table 1.** The IC_50_ Values for the Samples in the B-CPAP, SW and Hu02 Cell Lines Were Determined Following a 24-hour Incubation Period ^a^

Sample	IC_50_ (µg/mL) ^b^
**Ep on B-CPAP**	7.21 ± 0.558
**Et on B-CPAP**	77.51 ± 0.798
**RA on B-CPAP**	3.01 ± 0.838
**VA on B-CPAP**	407.29 ± 0.522
**Ep on SW**	92.9 ± 0.704
**Et on SW**	43.62 ± 0.801
**RA on SW**	1.83 ± 0.744
**VA on SW**	584.32 ± 0.931
**Ep on Hu02**	914.96 ± 0.998
**Et on Hu02**	408.31 ± 1.002
**RA on Hu02**	~941.32 ± 0.98
**VA on Hu02**	1833.11 ± 0.65

Abbreviations: Ep, epirubicin; Et, etoposide; RA, retinoic acid; VA, valproic acid.

^a^ Values are expressed as mean ± standard error of the mean (SEM).

^b^ The IC_50_ values were determined using Graph Pad Prism 5.0 program with a 95% confidence interval.

### 4.2. The Impact of Single and Combined Drug Concentrations on the Induction of Apoptosis and Necrosis

**Figure 1.**
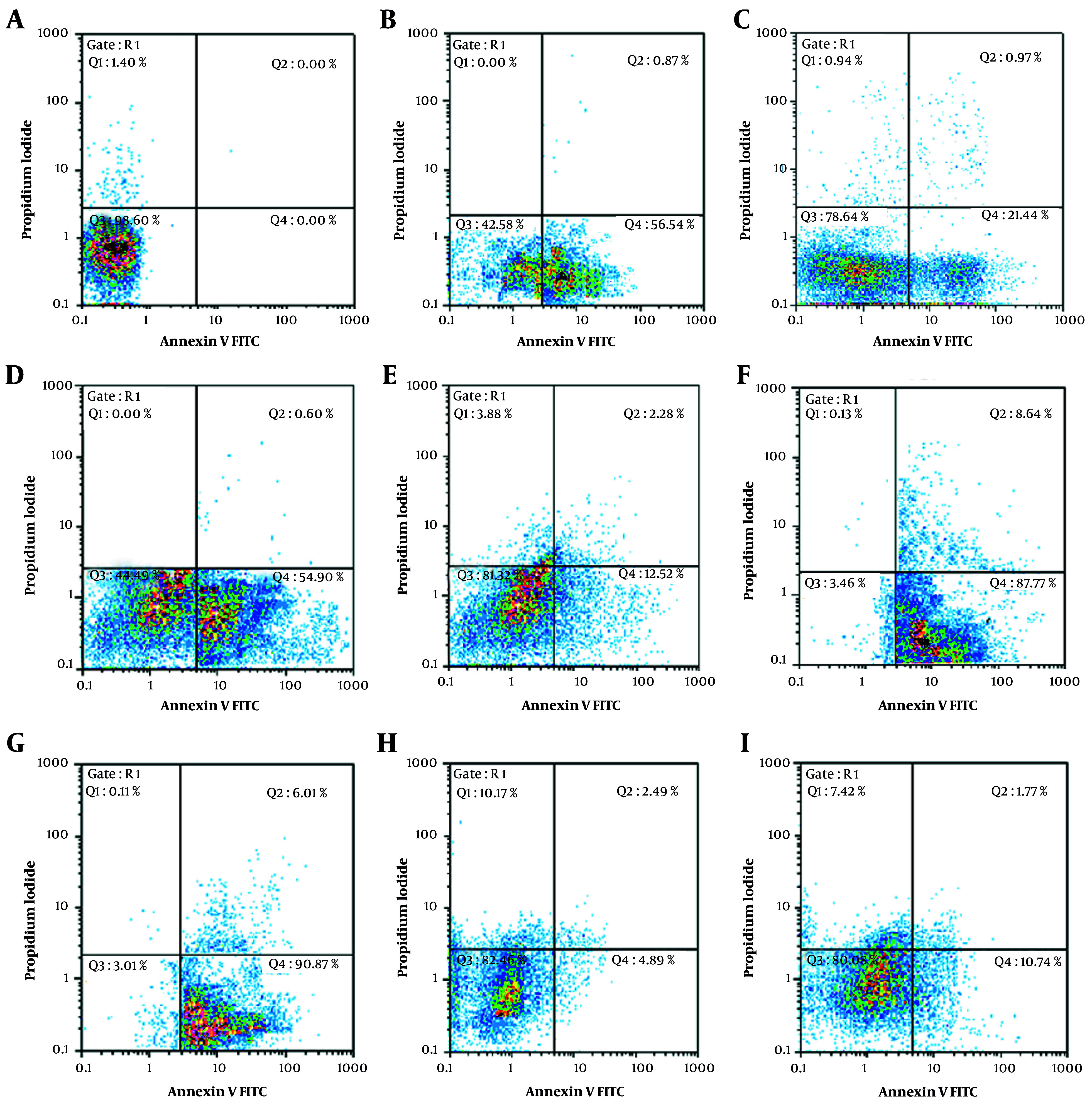
Annexin V-FITC vs. propidium iodide (PI) quantitation of B-CPAP cells in control and test groups including: A, non-treated (RPMI as negative control); B, epirubicin (Ep; IC_50_ dose); C, Ep (1/5 IC_50_ dose); D, etoposide (Et; IC_50_ dose); E, Et (1/5 IC_50_ dose); F, retinoic acid (RA; IC_50_ dose) in combination with Ep (1/5 IC_50_ dose); G, RA (IC_50_ dose) in combination with Et (1/5 IC_50_ dose); H, valproic acid (VA; IC_50_ dose) in combination with Ep (1/5 IC_50_ dose); and I, VA (IC_50_ dose) in combination with Et (1/5 IC_50_ dose).

**Figure 2.**
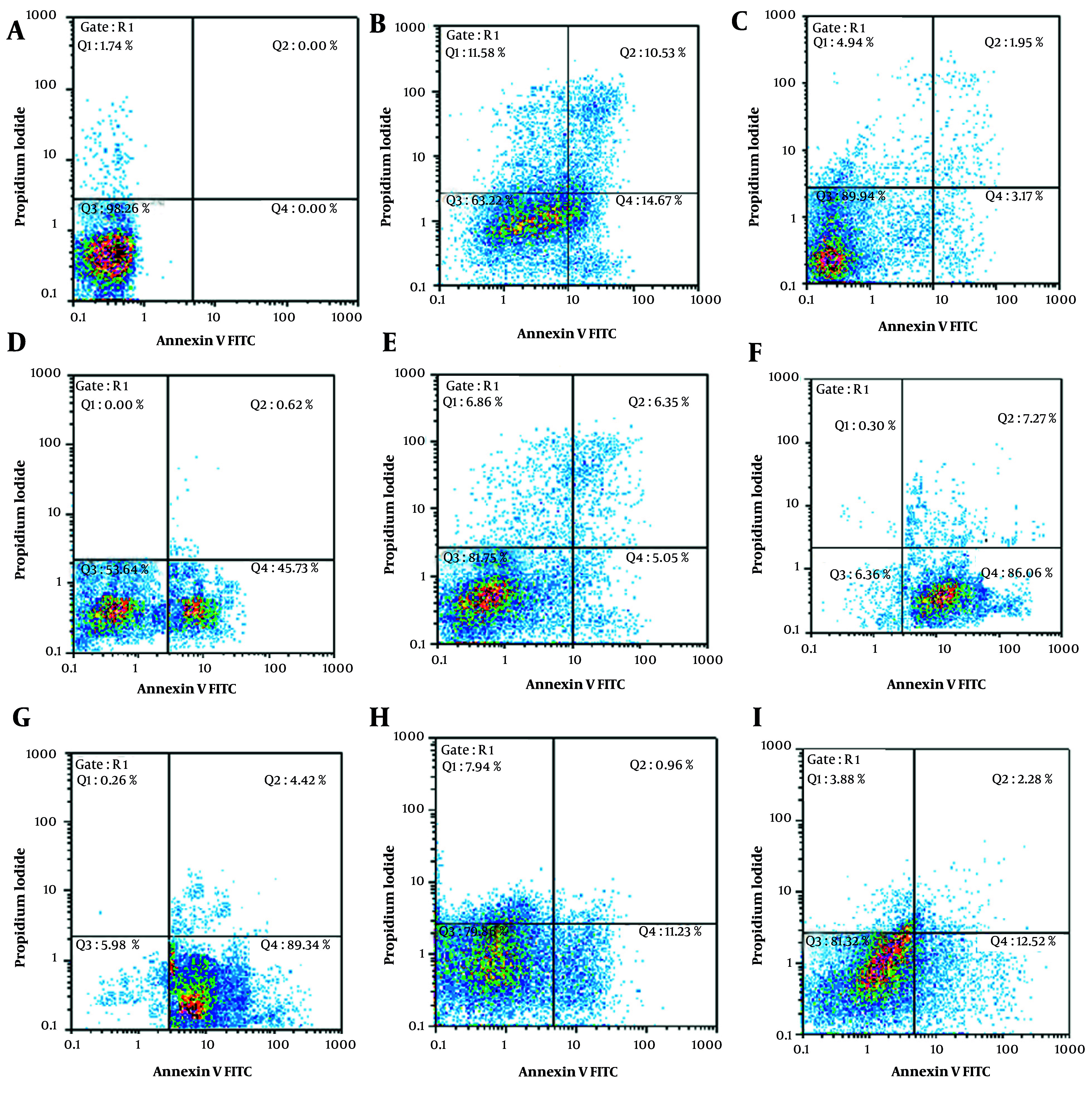
Annexin V-FITC vs. propidium iodide (PI) quantitation of SW cells in control and test groups including: A, non-treated (RPMI as negative control); B, epirubicin (Ep; IC_50_ dose); C, Ep (1/5 IC_50_ dose); D, etoposide (Et; IC_50_ dose); E, Et (1/5 IC_50_ dose); F, retinoic acid (RA; IC_50_ dose) in combination with Ep (1/5 IC_50_ dose); G, RA (IC_50_ dose) in combination with Et (1/5 IC_50_ dose); H, valproic acid (VA; IC_50_ dose) in combination with Ep (1/5 IC_50_ dose); and I, VA (IC_50_ dose) in combination with Et (1/5 IC_50_ dose).

Further analysis of apoptosis induction, shown in Figures 1 and 2, revealed that RA at IC_50_ concentration significantly enhanced early apoptosis when combined with low-dose Et/Ep (1/5 IC_50_) compared to Et/Ep treatment alone (P < 0.05). In contrast, VA showed no significant enhancement of apoptotic effects when combined with these drugs.

### 4.3. The Impact of Individual and Combined Drug Concentrations on the TC Cell Cycle

**Figure 3.**
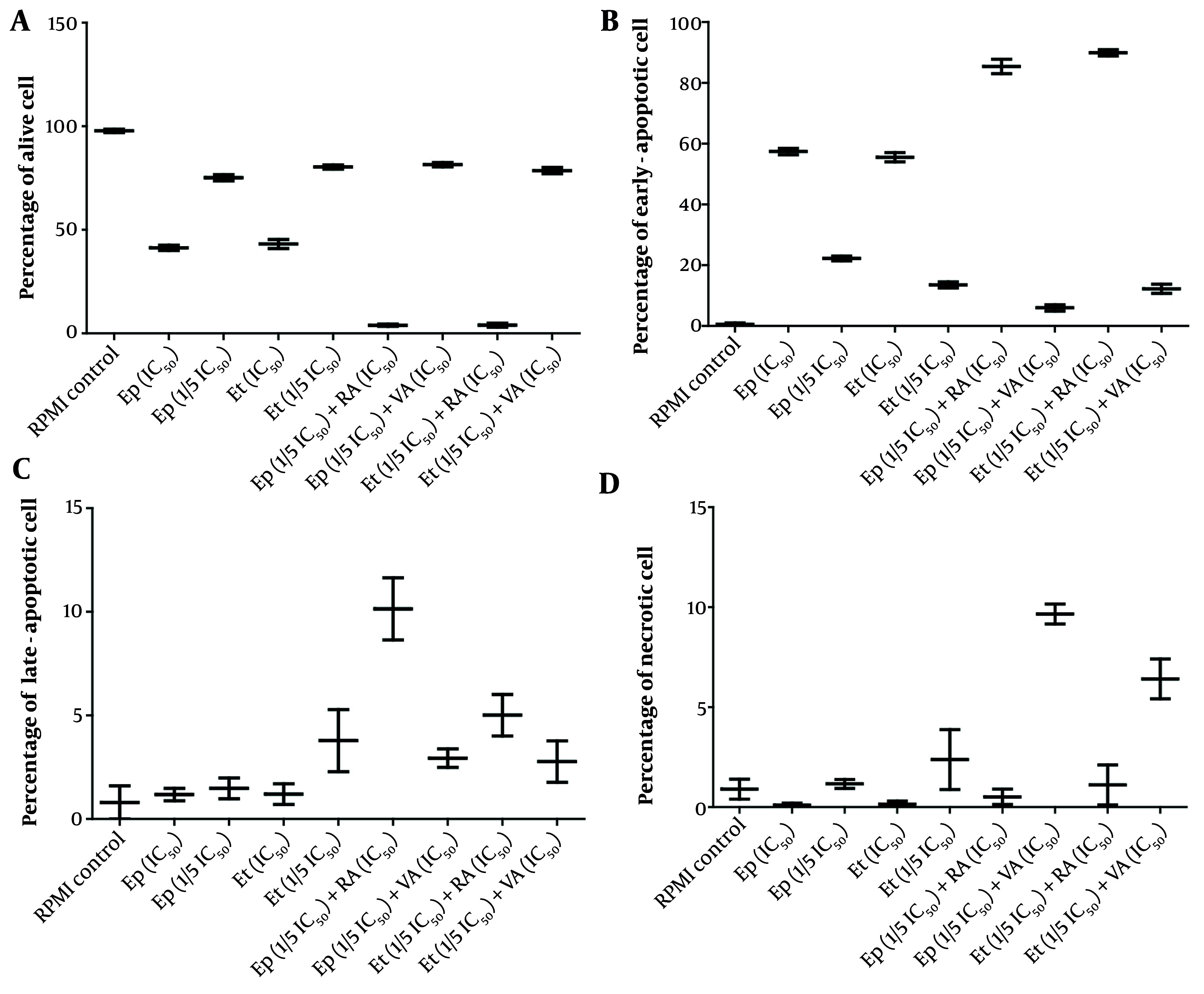
The cytotoxic effects of epirubicin (Ep) and etoposide (Et) were compared in single doses, as well as in combination with retinoic acid (RA) and valproic acid (VA) on percentage of A, alive; B, early-apoptotic; C, late-apoptotic; and D, necrotic cell in B-CPAP cells after 48 hours of incubation.

**Figure 4.**
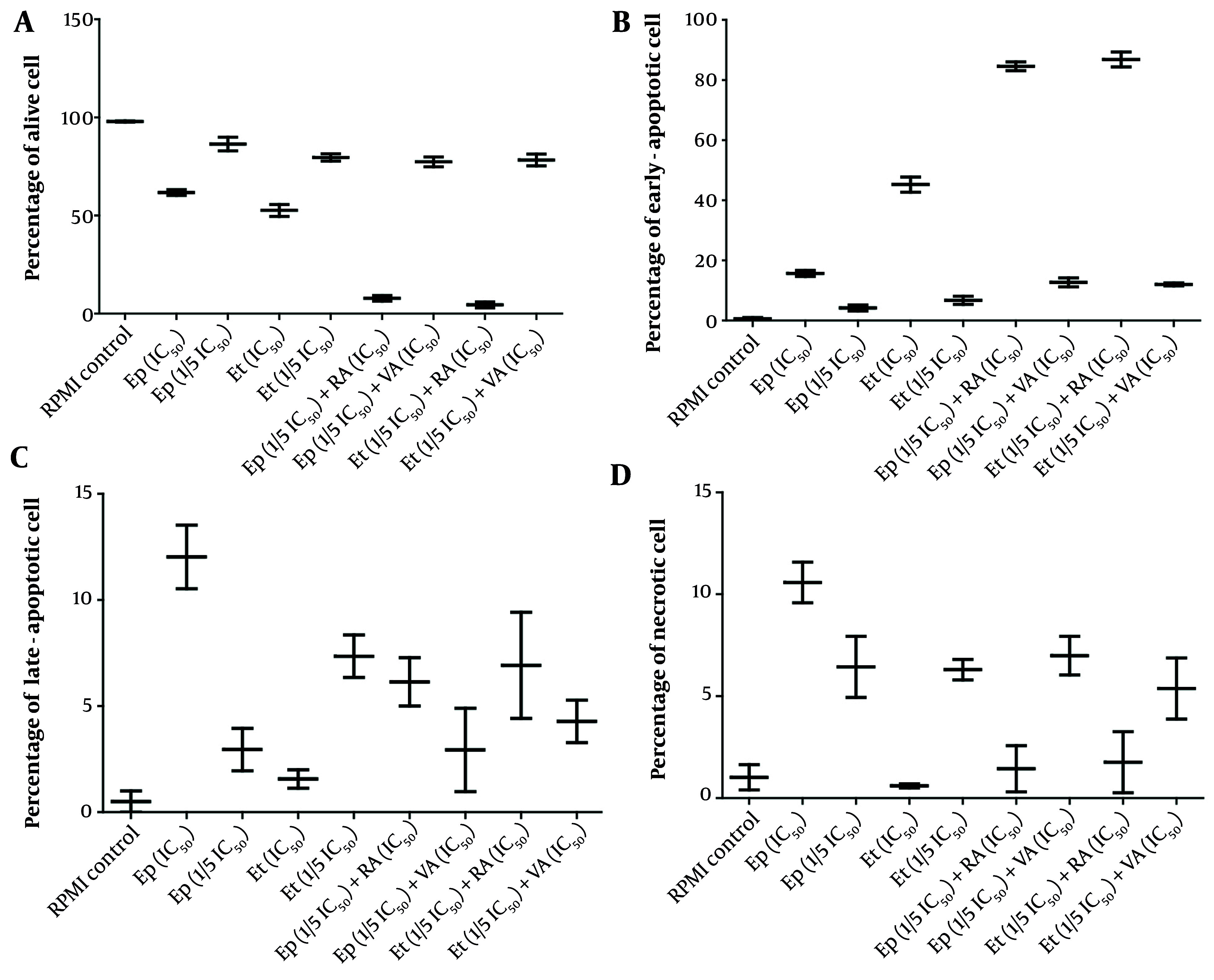
The cytotoxic effects of epirubicin (Ep) and etoposide (Et) were compared in single doses, as well as in combination with retinoic acid (RA) and valproic acid (VA), on percentage of A, alive; B, early-apoptotic; C, late-apoptotic; and D, necrotic cell in B-SW cells after 48 hours of incubation.

**Figure 5.**
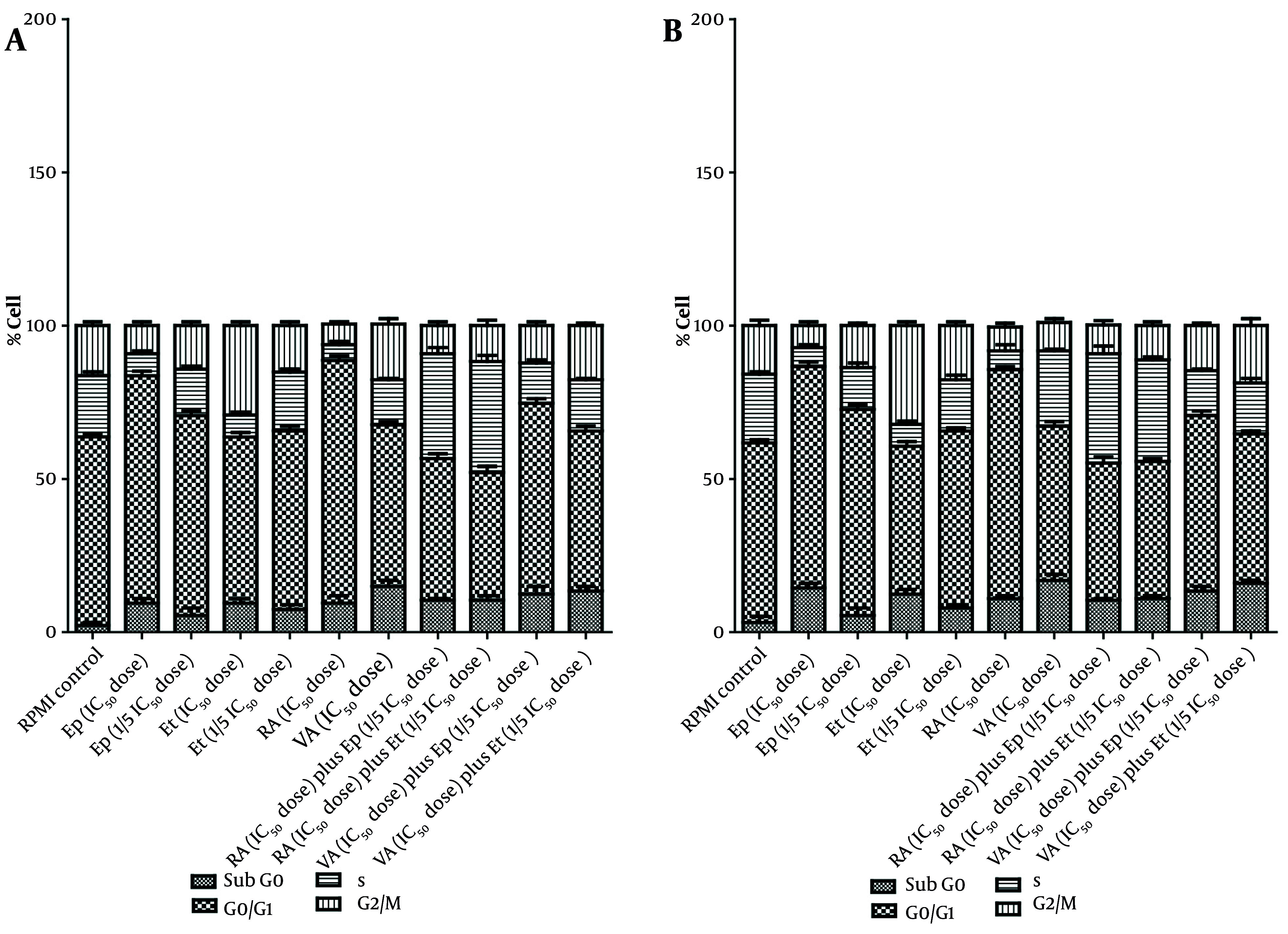
The impact of epirubicin (Ep) and etoposide (Et) at different concentrations (IC_50_ and 1/5 IC_50_ doses), both alone and in combination with retinoic acid (RA) and valproic acid (VA), on the distribution of cell cycle in the A, B-CPAP and B, SW cell lines was assessed after a 48-hour incubation period [the data is expressed as the mean ± standard deviation; significant differences (* P < 0.05) were observed compared to the control group treated with RPMI].

Cell cycle analysis, presented in Figures 3 - 5, demonstrated distinct phase-specific arrest patterns: The Ep (IC_50_) induced G0/G1 arrest, Et (IC_50_) caused G2/M arrest, and RA alone triggered G0/G1 phase arrest. Notably, the combination of RA (IC_50_) with low-dose Et/Ep resulted in significant S-phase arrest (P < 0.05), while VA combinations showed minimal effects on cell cycle distribution.

### 4.4. The Impact of Individual and Combined Drug Concentrations on Cancer Cell Migration

**Figure 6.**
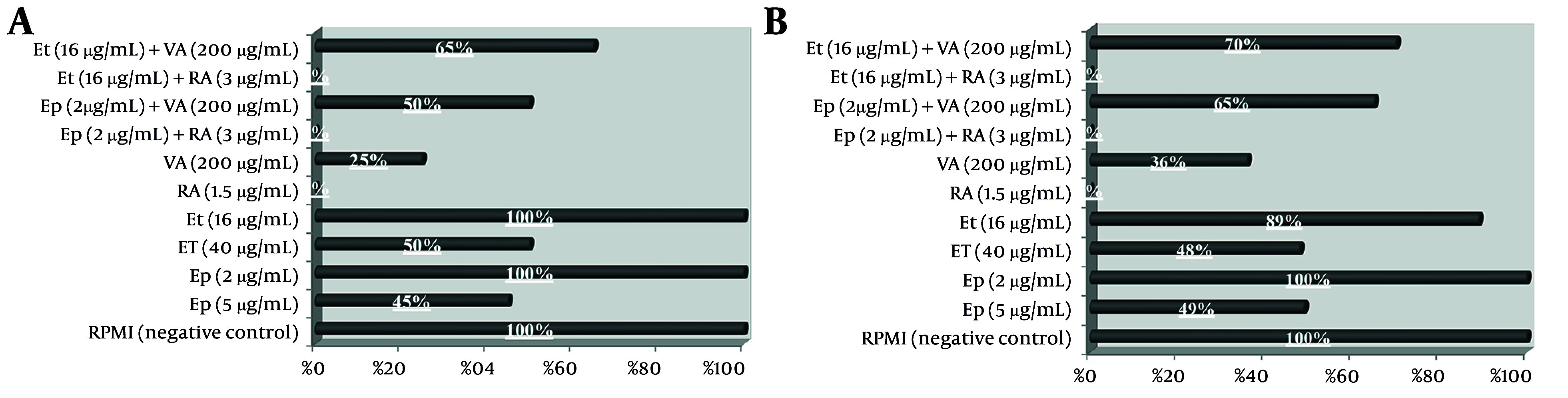
The migration percentage of A, B-CPAP and B, SW cells was determined using a scratch wound healing assay after treatment with epirubicin (Ep), etoposide (Et), retinoic acid (RA), and valproic acid (VA) individually and in combination.

The impact on cell migration, evaluated through scratch wound assays and illustrated in Figure 6, showed that single-agent treatments with Et/Ep at 1/2 IC_50_ reduced migration by approximately 50%, while VA and RA at the same concentration achieved 70% and complete (100%) inhibition, respectively. Combination treatments revealed that RA maintained complete inhibition (100%) when combined with low-dose Et/Ep (1/5 IC_50_), whereas the VA combination showed reduced efficacy (40% inhibition).

## 5. Discussion

Our study provides substantial preclinical evidence that RA-based combinations represent a transformative approach for overcoming key therapeutic challenges in thyroid cancer. While previous studies have established the general anticancer properties of RA and VA (21-23), our systematic comparison reveals three critical advances: (1) The RA's superior synergy with conventional agents (CI < 1), (2) its unique cell cycle modulation capabilities when combined with Et/Ep (S-phase accumulation vs. The VA's minimal effects), and (3) a 10-fold therapeutic window exceeding VA's 2-fold margin — addressing the urgent clinical need for less toxic regimens (5, 6, 24). These findings directly challenge the prevailing assumption that HDAC inhibitors like VA should be prioritized for thyroid cancer combination therapies (14, 21).

The mechanistic divergence between RA and VA combinations is particularly striking. Our apoptosis assays demonstrate that RA enhances early apoptosis by 3-fold compared to VA when combined with low-dose chemotherapy (P < 0.01), corroborating recent work on RA's pro-apoptotic signaling (8, 9) while contradicting earlier reports favoring VA's epigenetic effects (14). The complete inhibition of migration by RA (100% vs. VA's 40 - 70%) (22) further suggests RA may uniquely target both proliferative and metastatic pathways — a finding with immediate implications for anaplastic thyroid cancer (ATC) treatment where metastasis drives mortality (2, 15).

Our cell cycle data reveal an unexpected therapeutic opportunity: The RA monotherapy induced G0/G1 arrest, but RA-chemotherapy combinations caused S-phase accumulation. This paradoxical shift, not observed with VA, implies that RA may sensitize cells to S-phase targeting agents — a hypothesis supported by recent work on cell cycle synchronization (25) but previously unexplored in thyroid cancer. This finding directly informs the ongoing debate about optimal drug sequencing in combination therapy (23, 24), suggesting RA pretreatment could enhance subsequent chemotherapeutic efficacy.

The clinical translation potential of these findings is underscored by RA's favorable toxicity profile in normal Hu02 cells (10-fold window vs. cancer cells). This addresses two major limitations of current thyroid cancer therapies: Doxorubicin's cardiotoxicity and cisplatin's nephrotoxicity (21). Our results align with emerging paradigms in precision oncology that prioritize Therapeutic Index over absolute potency (6), particularly relevant for differentiated thyroid cancers where preserving quality of life is paramount (2, 12).

This study suggests three significant implications for further clinical investigations. First, RA's dual inhibition of proliferation and migration suggests its potential to reverse epithelial-mesenchymal transition, expanding upon previous redifferentiation hypotheses while offering direct relevance for radioiodine-refractory cases. Second, RA's lipophilic nature combined with our compelling migration inhibition data indicates its potential efficacy against CNS metastases — a critical unmet need in anaplastic thyroid cancer treatment. Third, the observed S-phase accumulation effect with RA-chemotherapy combinations provides a robust mechanistic foundation for optimizing treatment sequencing, addressing a key gap in combination therapy design for thyroid malignancies.

While these advances are promising, we identify two key limitations that define future research directions: (1) The need to validate RA's molecular targets through proteomic analysis [particularly RARβ isoforms (9,15)], and (2) the requirement for in vivo pharmacokinetic studies to optimize dosing schedules. The absence of synergy with VA suggests HDAC inhibition may be less critical than retinoid signaling in thyroid cancer — a hypothesis requiring testing in RAR-knockout models.

This study shifts the paradigm for thyroid cancer combination therapy by demonstrating RA's unique advantages over VA across multiple therapeutic dimensions. By establishing RA's synergistic potency, favorable toxicity profile, and novel cell cycle effects, we provide a preclinical foundation for clinical trials testing RA-chemotherapy combinations — particularly for radioiodine-refractory and anaplastic subtypes. These findings challenge current treatment algorithms (15, 26) and offer a template for evaluating natural product-derived agents in precision oncology (5, 6). Future work should focus on translating these mechanistic insights into optimized therapeutic sequences and biomarker-driven patient selection.

## Data Availability

The dataset presented in the study is available on request from the corresponding author during submission or after publication.
